# Inclisiran: How Widely and When Should We Use It?

**DOI:** 10.1007/s11883-022-01056-0

**Published:** 2022-07-25

**Authors:** Angela Pirillo, Alberico Luigi Catapano

**Affiliations:** 1 Center for the Study of Atherosclerosis, E. Bassini Hospital, Cinisello Balsamo, Milan, Italy; 2 Center for the Study of Dyslipidaemias, IRCCS MultiMedica, Sesto S. Giovanni, Milan, Italy; 3 Department of Pharmacological and Biomolecular Sciences, University of Milan, Milan, Italy

**Keywords:** Inclisiran, Low-density lipoprotein cholesterol, Secondary prevention, Primary prevention, Atherosclerotic cardiovascular disease

## Abstract

**Purpose of Review:**

Plasma levels of LDL cholesterol (LDL-C) are causally associated with cardiovascular risk. Reducing LDL-C results in a decreased incidence of cardiovascular events, proportionally to the absolute reduction in LDL-C. The inhibition of proprotein convertase subtilisin kexin 9 (PCSK) is a highly effective and safe approach to reducing LDL-C levels. In this review, we discuss the available data on the efficacy and safety of inclisiran, a siRNA targeting PCSK9 and propose a clinical profile for the patients who can benefit the most from this approach.

**Recent Findings:**

Inclisiran is a small interfering RNA targeting the mRNA of PCSK9 specifically in the liver, owing to the conjugation with triantennary N-acetylgalactosamine. Randomized clinical trials have shown that inclisiran provides robust and durable reductions of PCSK9 and LDL-C levels, with a dosing schedule of once every 6 months after the initial and 3-month doses. These effects are consistent in different categories of patients, including patients with atherosclerotic cardiovascular disease and/or risk equivalent or patients with heterozygous familial hypercholesterolaemia. Ultimately the administration schedule may improve patients’ compliance given also the favourable safety profile of the drug.

**Summary:**

Completion of ongoing outcome clinical trials will provide information on both the expected clinical benefit and the safety of inclisiran administered for longer.

## Introduction

The causal association between plasma levels of LDL cholesterol (LDL-C) and cardiovascular (CV) risk has been unequivocally established [1, 2]. A large number of randomized clinical trials has proven that reducing LDL-C levels results in a reduced incidence of cardiovascular events and that the larger (and earlier) the LDL-C reduction achieved, the greater the clinical benefit [1, 3–6]. In the last decades, the availability of drugs effectively lowering LDL-C and atherosclerotic cardiovascular disease has increased significantly. So far, the combination of drugs with complementary mechanisms of action allowed to achieve low LDL-C levels, with evidence supporting CV clinical benefit that extends monotonically with LDL-C lowering without reaching a plateau, even for very low LDL-C (< 30 mg/dL) [7–9]. This evidence has prompted a change in the LDL-C goals in guidelines such as those from ESC/EAS, which now recommend more stringent goals for high and very high-risk patients [10].

Among the most recent LDL-C-lowering approaches, targeting proprotein convertase subtilisin kexin 9 (PCSK9) was shown to be a highly effective and safe approach for reducing LDL-C. The development and approval of two monoclonal antibodies (mAbs, evolocumab and alirocumab) and, more recently, a gene-silencing agent (inclisiran) have profoundly affected the landscape of LDL-C-lowering and cardiovascular prevention. The latter is an innovative approach to managing hypercholesterolaemia and CV prevention, with a dosing schedule considerably different from that of common cholesterol-lowering drugs and potentially advantageous in terms of adherence and compliance to therapy. In this review, we will discuss the results of the available clinical trials assessing the efficacy and safety of inclisiran and propose a clinical profile for the patients who might benefit the most from this new approach.

## PCSK9

PCSK9 is a serine protease endowed with a relevant role in the modulation of hepatic LDL receptor (LDLR) expression. After secretion in the bloodstream, PCSK9 binds to LDLR, triggering the internalization of the complex, a conformational change of LDLR, and the degradation of the latter within lysosomes [11–13]. Genetics has provided strong support for the role of PCSK9 in atherosclerosis: loss-of-function mutations in *PCSK9* are associated with lower levels of circulating LDL-C and a reduced risk of coronary heart disease [14–19]; whereas gain-of-function mutations, determining elevated LDL-C levels, cause familial hypercholesterolaemia (FH) and increase the risk of premature cardiovascular disease [20–24]. These observations have suggested PCSK9 as a target for controlling hypercholesterolaemia, leading to the rapid development and approval of evolocumab and alirocumab, two monoclonal antibodies targeting circulating PCSK9, and inclisiran, a small interfering RNA targeting hepatic PCSK9 synthesis. A large number of clinical trials have investigated the lipid-lowering effect and clinical benefit of mAbs to PCSK9 in many categories of patients with hypercholesterolaemia or established atherosclerotic cardiovascular disease (ASCVD), proving their efficacy and safety [25–27].

## How Do the Approved Strategies Targeting PCSK9 Differ?

Inclisiran is a double-stranded, small interfering RNA (siRNA) that, by targeting the mRNA of PCSK9 specifically in the liver, inhibits the hepatic synthesis of PCSK9 protein. The liver-specificity of inclisiran is provided by the conjugation on the sense strand with triantennary N-acetylgalactosamine carbohydrate (GalNAC), which facilitates its uptake through the asialoglycoprotein receptor, highly expressed in hepatocytes, but not in muscles. Within hepatocytes, inclisiran is incorporated into an RNA-induced silencing complex (RISC) which ensures a long-lasting and robust silencing of hepatic PCSK9 mRNA [13].

Although resulting in the inhibition of the same protein, the specific mechanisms of action of inclisiran and mAbs may have profound biological implications.

PCSK9 is expressed at high levels in the liver, which contributes to the bulk of circulating PCSK9. Other tissues express PCSK9, such as the kidney and intestine, and thus PCSK9 is expected to have a role beyond the liver. Based on this observation, a major concern arising from the use of a mAb targeting circulating PCSK9 is the possibility that it might interfere with processes not directly related to the control of plasma LDL-C levels. In this regard, genetic PCSK9 deficiency not only increases the risk of developing diabetes [28] or ectopic fat accumulation [29], but also reduces insulin secretion and promotes glucose intolerance [30], and affects cardiac metabolism and function [31]. However, it has been shown that the local PCSK9 deficiency rather than circulating PCSK9 has a role in defining these phenotypes, which should relieve mAbs for possible “off-target” effects beyond LDL-C level control [30, 31]. As opposed to mAbs, inclisiran specifically targets hepatic PCSK9 synthesis, thus reducing circulating PCSK9, but does not interfere with the synthesis of PCSK9 in non-hepatic tissues.

Furthermore, although these biologics are all administered via subcutaneous injection, mAbs need to be administered once or twice a month, whereas inclisiran is administered every 6 months after initial baseline and 3-month doses, which may represent an undeniable opportunity to improve adherence to therapy.

## Results From the ORION Program

Following the results of preclinical studies showing that targeting PCSK9 with a siRNA reduced significantly plasma PCSK9 and LDL-C levels [32], a phase 1 study showed that inclisiran reduced PCSK9 (up to 70%) and LDL-C (up to 40%) for at least 6 months in healthy volunteers with baseline LDL-C ≥ 100 mg/dL [33]. The ORION clinical development program consists of phase 2 and 3 clinical trials, some of which have been completed. The ORION-1 is a phase 2 trial in which 501 patients at high CV risk and elevated LDL-C levels (> 70 mg/dL for patients with a history of ASCVD or > 100 mg/dL for patients without a history of ASCVD) were randomized to receive a single dose or two doses (at days 1 and 90) of inclisiran or placebo in addition to their current lipid-lowering therapy (LLT) [34]. On day 180, patients achieved 27.9 to 41.9% LDL-C level reductions with the single-dose regimen and 35.5 to 52.6% with the two-dose regimen. A mean absolute LDL-C reduction of 64.2 mg/dL was reported at day 180 in patients who received two 300-mg doses of inclisiran, a reduction that persisted up to day 240; 54% of these patients experienced a ≥ 50% reduction [34]. Total cholesterol, non-HDL-C, and apoB were all significantly and dose-dependently reduced by inclisiran; Lp(a) was also reduced up to 18.2% with the single-dose regimen and up to 25.6% with the two-dose regimen [34]. A 50% LDL-C reduction was maintained for at least 6 months after 2 doses of 300 mg inclisiran; patients maintained an LDL-C reduction of at least 39 mg/dL (1 mmol/L) from baseline for a median of 6 to 9 months with a single dose and from 5 to 10 months with 2 doses of inclisiran [35]. These results suggest a long-lasting effect of inclisiran. The ORION-3 study is the open-label extension study of ORION-1 assessing the effect of long-term dosing of 300 mg inclisiran administered twice yearly for up to 4 years (NCT03060577). An interim analysis at ~ 22 months showed a persistent 51% LDL-C reduction, with ~ 60 mg/dL time-averaged lowering in LDL-C [36]. The final results are expected shortly.

The phase 3 trials ORION-9, -10, and -11 assessed the efficacy and safety of inclisiran in patients with heterozygous FH (HeFH), ASCVD and/or risk equivalent receiving maximally tolerated lipid-lowering therapy. HeFH patients who received 300 mg inclisiran on days 1, 90, 270 and 450 had significant reductions in LDL-C levels compared with patients receiving placebo: at day 510, a between-group percent difference of 47.9% in LDL-C levels (time-averaged 44.3%) was observed, corresponding to a between-group absolute difference of 68.9 mg/dL (time-averaged 62.6 mg/dL) [37]. This robust reduction was observed across all types of genetic defects determining FH and was similar to that observed in patients treated with mAbs to PCSK9 [37]. Unlike statins, and in agreement with the effect observed with mAbs to PCSK9, inclisiran reduced Lp(a) levels by 17.2% (placebo-adjusted) from baseline [37]. ORION-10 and -11 trials showed similar reductions in hypercholesteraemic patients with ASCVD or ASCVD risk equivalent, with LDL-C cholesterol level reductions of 52.3% and 49.9% at day 510 (time-averaged 53.8% and 49.2%), and absolute reductions of 54.1 mg/dL and 51.9 mg/dL (time-averaged 53.3 mg/dL and 48.9 mg/dL), respectively [38]. Inclisiran reduced Lp(a) by 25.6% and 18.6% in ORION-10 and -11, respectively [39••]. ORION-8 is an open-label extension trial of the phase 3 trials ORION-9, -10, and -11, designed to assess the effect of long-term dosing of 300-mg inclisiran given twice yearly to day 990 (NCT03814187), with an estimated completion date in August 2023. To prove the clinical benefit of inclisiran, the ORION-4 trial will assess whether PCSK9 silencing safely lowers the risk of major atherosclerotic cardiovascular events in ≥ 15,000 patients with pre-existing ASCVD during a median treatment duration of 5 years (NCT03705234). The estimated primary completion date is December 2024. The VICTORION-2P is a pivotal phase III trial that will evaluate the benefits of inclisiran on major adverse cardiovascular events in participants with established cardiovascular disease in addition to their well-tolerated lipid-lowering therapy (high-intensity statin with or without ezetimibe), with a follow-up of up to 72 months (NCT05030428).

Inclisiran has also been tested in HoFH patients in the ORION-2 pilot study including 4 individuals (2 homozygotes and 2 compound heterozygotes); although all of them had robust and durable reductions in PCSK9 levels, only 3 achieved reductions in LDL-C (although smaller in proportion than those observed in HeFH or non-FH patients) [40]. A fourth patient showed no reduction in LDL-C, despite bearing the same genetic defect as a responsive patient, highlighting the great complexity of this pathological condition. The ongoing phase 3 ORION-5 trial (NCT03851705) is currently assessing the efficacy of inclisiran in a larger sample of HoFH patients. Based on the limited experience on younger patients, which should indeed be treated at the earliest and as safely as possible to improve their long-term quality of life, two trials will evaluate the efficacy and safety of inclisiran in adolescents (aged 12–17 years) with homozygous FH (ORION-13) or heterozygous FH (ORION-16); these trials include a 1-year double-blind study period (inclisiran vs. placebo) and 1-year open-label treatment with inclisiran [41]. Results coming from these trials, together with the advantage of an infrequent, twice-yearly administration schedule, may pave the way for an additional effective, lifelong LDL-C-lowering option in patients with FH.

Based on the results obtained in completed trials from this program, inclisiran has been approved by US and European regulators for the treatment of adults with primary hypercholesterolaemia (heterozygous familial and non-familial) or mixed dyslipidaemia, in combination with a statin or statin with other lipid-lowering therapies in patients unable to reach LDL-C goals with the maximum tolerated dose of a statin, or alone or in combination with other lipid-lowering therapies in patients who are statin-intolerant, or for whom a statin is contraindicated.

## How Widely and When Inclisiran Should Be Used?

Taking for granted that inclisiran reduces persistently LDL-C levels, and while awaiting the results of ongoing outcome trials, which will tell whether the use of inclisiran provides a consistent clinical benefit, it remains to be addressed how widely and when this new cholesterol-lowering approach should be used. Some aspects can limit its wider clinical use, including high cost, subcutaneously administration route, limited accessibility, and lack of long-term clinical outcome and safety data. Based on a large number of observations from randomized clinical trials, prospective cohort studies, and Mendelian randomization studies, that have established a log-linear relationship between LDL-C and ASCVD risk and that LDL-C-lowering always results in a reduced CV risk independently of the LLT used, it is conceivable that inclisiran will reduce the CV risk proportionally to the entity of LDL-C absolute reduction.

Randomized clinical trials with a median 5-year follow-up of statin therapy have established that each mmol/L LDL-C reduction produces a 22% proportional reduction in the risk of major cardiovascular events [1, 3] (a benefit that, however, can be even higher in long-term treatments, as LDL-C reduction tends to be progressively less over time). Results from outcome trials have shown that anti-PCSK9 mAb-based therapies reduce LDL-C levels by approximately 60%, which translated into a 15% relative risk reduction in the primary endpoint [5, 6]. Taking into account the shorter follow-up of these last trials (2.2 years in Fourier and 2.8 years in Odyssey Outcomes), it appears that mAbs to PCSK9 reduce the risk of CV events similarly to statins when analysed for the same duration of therapy [42] and that the clinical benefit of LDL-C-lowering is independent of the mechanism by which LDL-C is lowered. More importantly, the effect of LDL-C on the risk of ASCVD increases with increasing duration of exposure; 5 years of treatment with an LDL-C-lowering therapy reduces the relative CV risk of ASCVD by ~ 20–25% per mmol/L LDL-C reduction, while a 52-year exposure to lower LDL-C is expected to reduce ASCVD event risk by ~ 50–55% per mmol/L LDL-C reduction, as suggested by Mendelian randomization studies [42].

**Fig. 1:**
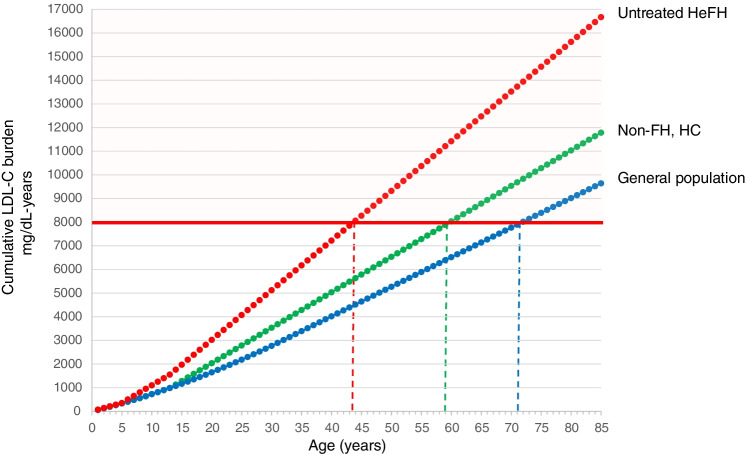
Cumulative effect of LDL-C in individuals with different LDL-C levels. Red dot line represents the cumulative LDL-C burden in untreated individuals with heterozygous familial hypercholesterolaemia; green dot line represents individuals developing hypercholesterolaemia in the teenage years; blue dot line represents individuals from the general population developing hypercholesterolaemia in their adulthood. The value of 8000 mg/dL-years represents the threshold after which the clinical evidence of atherosclerotic cardiovascular disease (ASCVD) becomes apparent

**Fig. 2:**
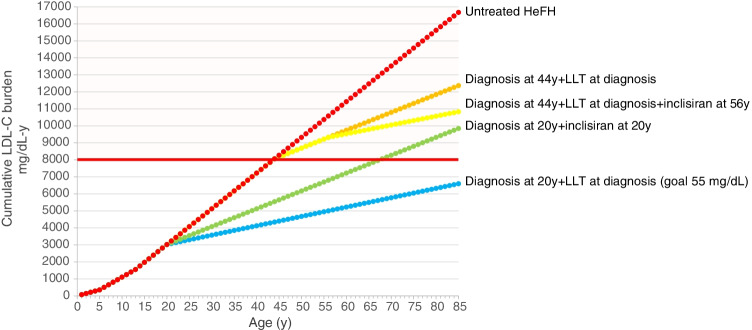
Cumulative effect of LDL-C in individuals affected by heterozygous FH with or without pharmacological therapy. Mean age of the first CV event in untreated HeFH is 44 years, that is also the mean age of HeFH diagnosis (FHSC registry). Red dots represent the cumulative LDL-C burden in untreated HeFH patients. Orange dots and yellow dots represent HeFH patients who start conventional lipid-lowering therapy alone (with an expected 50% reduction in LDL-C) or in combination with inclisiran (with a further 50% reduction). Green dots represent the curve of cumulative LDL-C burden in individuals diagnosed with HeFH earlier (at 20 years) and immediately starting inclisiran; blue dots represent the cumulative burden in HeFH individuals diagnosed at 20 years and treated with a combination therapy achieving the LDL-C goal of 55 mg/dL. HeFH, heterozygous familial hypercholesterolaemia; LDL-C, low-density lipoprotein cholesterol; LLT, lipid-lowering therapy

This last observation introduces the key notion of cholesterol burden i.e. the effect of the cumulative exposure to LDL-C over a lifetime, an estimate of cumulative LDL-C exposure obtained by multiplying the individual’s age by the LDL-C level. A theoretical threshold of LDL-C exposure after which coronary heart disease is more likely to occur has been set at ~ 8000 mg/dL-years; such a threshold is reached at different ages according to the individual LDL-C levels [43]. Thus, FH individuals with genetically determined high LDL-C levels from birth can exceed the threshold very early in life (especially if left untreated), not only compared with individuals of the general population developing hypercholesterolaemia in adulthood, but also compared with a non-FH, hypercholesterolaemic individual developing hypercholesterolaemia in the teenage years, secondary to genetics and/or non-optimal lifestyle habits (Fig. 1). This observation, on the other hand, implies that reducing LDL-C levels can substantially reduce the slope of the curve, thus shifting markedly the age at which the threshold is reached, and also underlines the importance of optimal LDL-C control starting early in life. The FHSC registry has reported that the median age at diagnosis of FH is ~ 44 years, with 59.5% of subjects taking lipid-lowering medications and having treated LDL-C levels 4.23 mmol/L (~ 164 mg/dL), and those without a lipid-lowering therapy (40.5%) having 5.43 mmol/L (~ 210 mg/dL) [44••]. In the ORION-9 trial, HeFH patients received inclisiran on top of their maximally tolerated LLT (mean LDL-C levels before inclisiran administration was ~ 150 mg/dL) at a mean age of 56 years [37]; despite the great reduction in LDL-C achieved in this trial, we may be reasonably assuming that these patients will have already reached the threshold of 8000 mg/dL-years (Fig. 2). On the other hand, if the FH diagnosis would be anticipated and patients would receive combination therapy with a 55 mg/dL LDL-C goal (as recommended by ESC/EAS guidelines) very early (at 20 years), the cumulative burden of LDL-C would be greatly reduced and the threshold will be reached much later in life (Fig. 2). In this context, combination therapy plays a crucial role in achieving the goal, and a twice-a-year inclisiran administration would ideally help in the lifelong management of young patients. Could a young HeFH patient be ideally treated only with inclisiran immediately after the diagnosis? Considering a 50% reduction in LDL-C levels, inclisiran could slow down the rate of progression of atherosclerosis to prevent or substantially delay the development of atherosclerotic plaques (Fig. 2).

Non-FH patients with documented ASCVD are considered at very high risk of CV events and mortality; for these patients, guidelines recommend very stringent LDL-C goals, which include an LDL-C < 55 mg/dL (a threshold that can be even lowered to < 40 mg/dL if experiencing recurrent events within 2 years) plus an at least 50% reduction in LDL-C from baseline. The feasibility of achieving such goals is strictly related to the baseline LDL-C levels and the individual response to cholesterol-lowering therapy. In most cases, combination therapy is required to reduce significantly LDL-C levels, starting from statin + ezetimibe and adding a PCSK9 mAb when required. The use of mAbs to PCSK9 has proven efficacy to reduce LDL-C levels and CV events, with a large percentage of treated patients (~ 80%) achieving a reduction of 50% or greater [45]; similarly, trials with inclisiran have shown a large proportion of patients experiencing reductions ≥ 50% [38]. Thus, this approach would allow this category of patients to more likely reach the recommended goal.

**Fig. 3:**
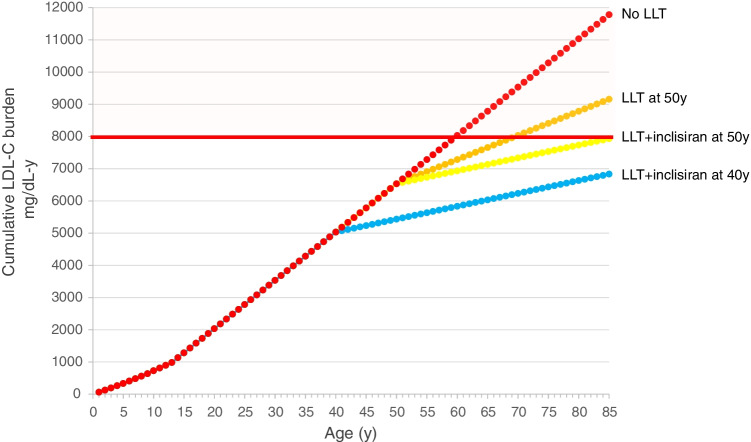
Cumulative effect of LDL-C in non-FH individuals developing hypercholesterolaemia early in life, with or without lipid-lowering therapy. Red dots represent the cumulative LDL-C burden in subjects developing hypercholesterolaemia early in life and not receiving any lipid-lowering treatment. Orange dots and yellow dots represent the same subjects starting conventional lipid-lowering therapy alone (with an expected 50% reduction in LDL-C) or in combination with inclisiran (with a further 50% reduction) at 50 years. Blue dots represent the curve in individuals starting lipid-lowering therapy in combination with inclisiran at 40 years. LDL-C, low-density lipoprotein cholesterol; LLT, lipid-lowering therapy

In the context of cardiovascular prevention, a non-FH individual with hypercholesterolaemia developing early in life will reach the threshold of 8000 mg/dL-years before the age of 60 (Fig. 3). Starting an LLT at 50 years with a conventional combination therapy that reduces LDL-C by 50% together with inclisiran (further 50% reduction) will reduce the progression of LDL-C exposure more than starting a conventional combination therapy without inclisiran (Fig. 3). Anticipating this approach by 10 years (i.e. at the age of 40 years) will theoretically shift substantially the age at which the threshold of 8000 mg/dL-years will be reached (Fig. 3).

**Fig. 4:**
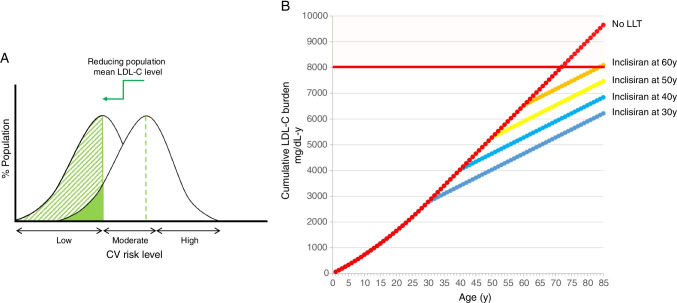
**a** Reduction of mean LDL-C levels at a population level. Reducing LDL-C levels in the whole population reduces the cardiovascular risk in a large number of individuals. **b** Cumulative effect of LDL-C in individuals developing hypercholesterolaemia later in life. Red dots represent the cumulative LDL-C burden in subjects developing hypercholesterolaemia later in life and not receiving any lipid-lowering treatment. The other curves represent the same individuals starting inclisiran at different ages. LDL-C, low-density lipoprotein cholesterol; LLT, lipid-lowering therapy

A relevant observation generated by a large meta-analysis of clinical trials with statins is that each 1 mmol/L LDL-C reduction produces the same relative CV risk reduction across all categories of cardiovascular risk, including individuals at the lowest risk [46]. In agreement with this finding, the Heart Outcomes Prevention Evaluation (HOPE)-3 trial showed that a simple approach with rosuvastatin 10 mg/day significantly reduces the risk of CV events in an intermediate-risk population without cardiovascular disease [47]. On the other hand, the absolute cardiovascular benefit deriving from an LLT depends on the individual risk: thus the same relative risk reduction may translate into a higher absolute risk reduction in individuals with high CV risk compared with a general population in primary prevention. However, the absolute number of prevented cardiovascular events may be numerically higher in the latter, due to the larger population size; thus, reducing LDL-C levels at a population level will shift the whole population into a lower risk category and will result in more individuals benefitting from this intervention than shifting a high-risk population into a lower risk category (Fig. 4a). Among the recent approaches under development for the cardiovascular prevention, vaccines against PCSK9 could be a better alternative than monoclonal antibodies [13, 48]; they would be less expensive, do not require frequent administrations, and could be part of a primary prevention strategy aimed at achieving optimal LDL-C levels among subjects who do not have clinical evidence of cardiovascular disease. To date, however, no data in humans are available with this approach. Although inclisiran is not a vaccine, its mechanism of action ensures infrequent administrations while maintaining high LDL-C-lowering efficacy; would it be conceivable to use inclisiran for primary prevention to reduce significantly the burden of LDL-C at a population level? In the absence of any type of LDL-C-lowering intervention, an individual with a mean LDL-C level ~ 120 mg/dL will reach the threshold of 8000 mg/dL-years at ~ 68 years (Fig. 4). Using an approach such as inclisiran, able to halve LDL-C levels, significantly will reduce the burden of LDL-C the more the earlier LDL-C level is reduced. The same reduction in LDL-C achieved at different ages will inevitably translate into a different clinical benefit, since “older” people will suffer from a residual risk deriving from a longer exposure to LDL-C and a consequent higher atherosclerotic plaque burden.

## Inclisiran in Perspective: Final Considerations

All the examples given so far are, of course, just speculative for a series of reasons. As first, although a large body of evidence has suggested that the clinical benefit of LDL-C-lowering is largely independent of the mechanism by which LDL-C is lowered and is strictly related to the absolute LDL-C reduction [1], we lack the demonstration that an inclisiran-based therapy reduces the incidence of cardiovascular events. To date, trials with inclisiran have enrolled only patients with ASCVD or HeFH under LLT (mostly statins with or without ezetimibe); thus, the demonstration that inclisiran might provide clinical benefits in monotherapy is still lacking. Furthermore, we need the demonstration that inclisiran is safe, especially for individuals who need a lifelong administration (such as FH) and before it can be conceived as a possible primary prevention intervention.

The twice-a-year administration schedule for inclisiran, on the other hand, is expected to improve greatly the adherence to therapy and maintain lower LDL-C levels for longer, which in turn is assumed to improve clinical outcomes. This represents an unquestionable advantage of this approach over other LDL-C-lowering drugs, most of which requires a daily administration and are characterized by low adherence to therapy, high rates of therapy discontinuation, reduced LDL-C goal attainment, and poorer clinical outcomes [49•]. A large proportion of patients discontinue also therapy with mAbs to PCSK9, which are injected typically every 2 or 4 weeks [50].

Clinical trials with inclisiran so far have not reported clinically relevant adverse effects [37, 38], but the duration was relatively short; ongoing outcome trials will provide long-term information on the safety of inclisiran. A possible caveat of the long-lasting activity of inclisiran is the insurgence of a severe adverse event making it difficult to rapidly reduce the exposition to the drug. Furthermore, inclisiran being a drug selectively taken up by hepatocytes, patients with some degree of hepatic impairment could present safety issues: a preliminary study in patients with mild or moderate hepatic impairment showed that inclisiran is effective and safe and required no dose adjustment [51]. A larger, long-term clinical trial is warranted to confirm the safety of inclisiran in patients with liver disease.
